# FOOD ASSISTANCE PROGRAMS AND REDUCED HOSPITALIZATIONS FOR OLDER ADULTS WITH DIABETES

**DOI:** 10.1093/geroni/igz038.535

**Published:** 2019-11-08

**Authors:** Monyca L Johnson, Sarah E Walsh

**Affiliations:** Eastern Michigan University, Ypsilanti, Michigan, United States

## Abstract

Diabetes is a an increasingly common and costly condition for older adults. Each year, as many as 1 in 3 Medicare dollars is spent to treat and manage diabetes and associated comorbidities for people with diabetes. To control health care spending in the US, it is imperative that we identify factors for reducing hospitalizations for these individuals. We used data from round five of the National Health and Aging Trends Study to identify predictors of hospitalization in the past 12 months for Medicare recipients ages 65 and older with diabetes. Previous research on the social determinants of health has demonstrated that social stressors like poverty and exposure to racism are associated with poorer health outcomes overall, but we did not find a statistically-significant association between race, gender or Medicaid dual-eligibility and hospitalization for our study population. Notably, receipt of SNAP benefits, Meals on Wheels services or other food assistance was associated with a 43% reduction in the risk of hospitalization in the past 12 months. As previous research has linked food insecurity with poorer medication adherence among individuals with Type II diabetes, food assistance programs appear to be an effective strategy for reducing hospitalizations associated with diabetes and its comorbidities.

